# A Facile Approach to Prepare Multiple Heteroatom-Doped Carbon Materials from Imine-Linked Porous Organic Polymers

**DOI:** 10.1038/s41598-018-22507-2

**Published:** 2018-03-09

**Authors:** Juan Yang, Min Xu, Jingyu Wang, Shangbin Jin, Bien Tan

**Affiliations:** Key Laboratory of Material Chemistry for Energy Conversion and Storage, Ministry of Education, School of Chemistry and Chemical Engineering, Huazhong University of Science and Technology, Wuhan, 430074 China

## Abstract

In this paper, we proposed a new strategy to prepare multiple heteroatom doped (*N*, *P*-doped) porous carbon materials with high surface area of ~1,535 m^2^ g^−1^ simply by pyrolysis of imine-linked porous organic polymers (POPs) synthesized *via* Schiff base condensation. The strategy is simple without any post-processing and various heteroatoms could be involved. Scanning electron microscopy, Raman spectra, Nitrogen gas adsorption-desorption, X-ray photoelectron spectroscopy have been used to characterize the morphology, the structure and the composition of the materials. The multiple heteroatom doped porous carbon materials also display high electrocatalytic performance as exampled by the application in oxygen reduction, which showed the catalyst favors 4-electron transfer during the process, along with superior stability and higher tolerance to methanol as compared to the Pt/C. These results indicate the present method is promising for the preparation of multi-heteroatom doped carbon materials in the application of electrocatalysis.

## Introduction

Porous organic polymers (POPs) are a type of organic polymers with large surface area, tunable skeleton and high stability^1,2^. They have attracted extensive attentions for broad applications in the fields of gas storage and separations, catalysis and energy storage^3–5^. There are many methodologies reported for the preparation of porous polymers^6–10^. Among them, Schiff base reaction is one of the most versatile ways due to the facile, one-pot, catalyst-free, quantitative synthesis^11^. Moreover, POPs can be constructed by using readily accessible and abundant monomers, which provides flexibility for the material and thus it is possible to design to achieve desirable porous properties (surface area, pore volume, pore width, etc.). Heteroatoms can be introduced as homogeneously distributed catalytic active sites in POPs without any post-modification^12–14^. Employing POPs as templates or precursors to synthesize nanoporous carbons becomes the hotspot in expanding application due to their outstanding advantages such as permanent nanoscale cavities and open channels^15,16^.

Recently, heteroatom-doped carbon materials have emerged as promising metal-free candidates for electrocatalysis applications, such as oxygen reduction reactions (ORR) in fuels cells^17–19^, advanced electrodes for supercapacitors^20,21^ and other clean-energy devices^22,23^. Comparing with metal-based materials such as Pt/C or non-precious-metal catalysts (e.g., Fe, Co, Ni, Mn, etc.), heteroatom-doped carbon materials are able to meet certain challenges, e.g. the dependence on high cost and scarcity noble metals^24,25^, a lack of long term stability^26,27^, low selectivity and poor durability^28,29^.

Homogeneous heteroatom doping, porous structure and surface area of heteroatom-doped carbon materials have synergistic effect on electrochemical properties of carbon materials. Both experimental and theoretical studies have proved that N atoms doping can effectively modify the electronic structures and surface chemical properties of the carbon networks, facilitating the electrochemical reaction on the carbon surface^30,31^. Co-doping carbon nanomaterials with N and other heteroatom (e.g., B, or S, or P, etc.) has been reported to further increase ORR activity due to the synergistic electronic effects of different dopants. The introduction of other heteroatom not only provides more active sites in carbon networks but also makes N-sites more catalytically active and efficient^32–35^. In addition, the rich porosity in carbon networks will bring about enlarged surface area, which increases the accessibility of reactants to surface dopants^36^. The construction of hierarchical porous architecture (including micro-, meso- and macropores) can facilitate the O_2_ and electrolyte ions transportation by shortening the diffusion pathways^37,38^. Finally, the large surface area of porous carbon materials which increases the accessibility of reactants to surface dopants, acts as another factor that affects the density of the catalytically active sites.

However, it is rarely reported to prepare multiple heteroatom-doped porous carbon materials using imine-linked porous organic polymers^39–41^. Although there were many other methods to prepare multiple heteroatom-doped carbon based on porous organic polymers, metal-organic frameworks or other materials, their synthesis was associated with complicated and environmentally harmful procedures, including hard templates involved^17^, post-modification^42^, noble metal-catalyzed reactions^43^, pyrolysis in NH_3_ environment^44^, or relatively low BET surface area^45^. Therefore, it is essential to develop more convenient and economic methods to construct multiple heteroatom-doped porous carbon materials with homogeneously distributed active sites, hierarchical porous architecture and large surface area to achieve high performance for application.

**Figure 1:**
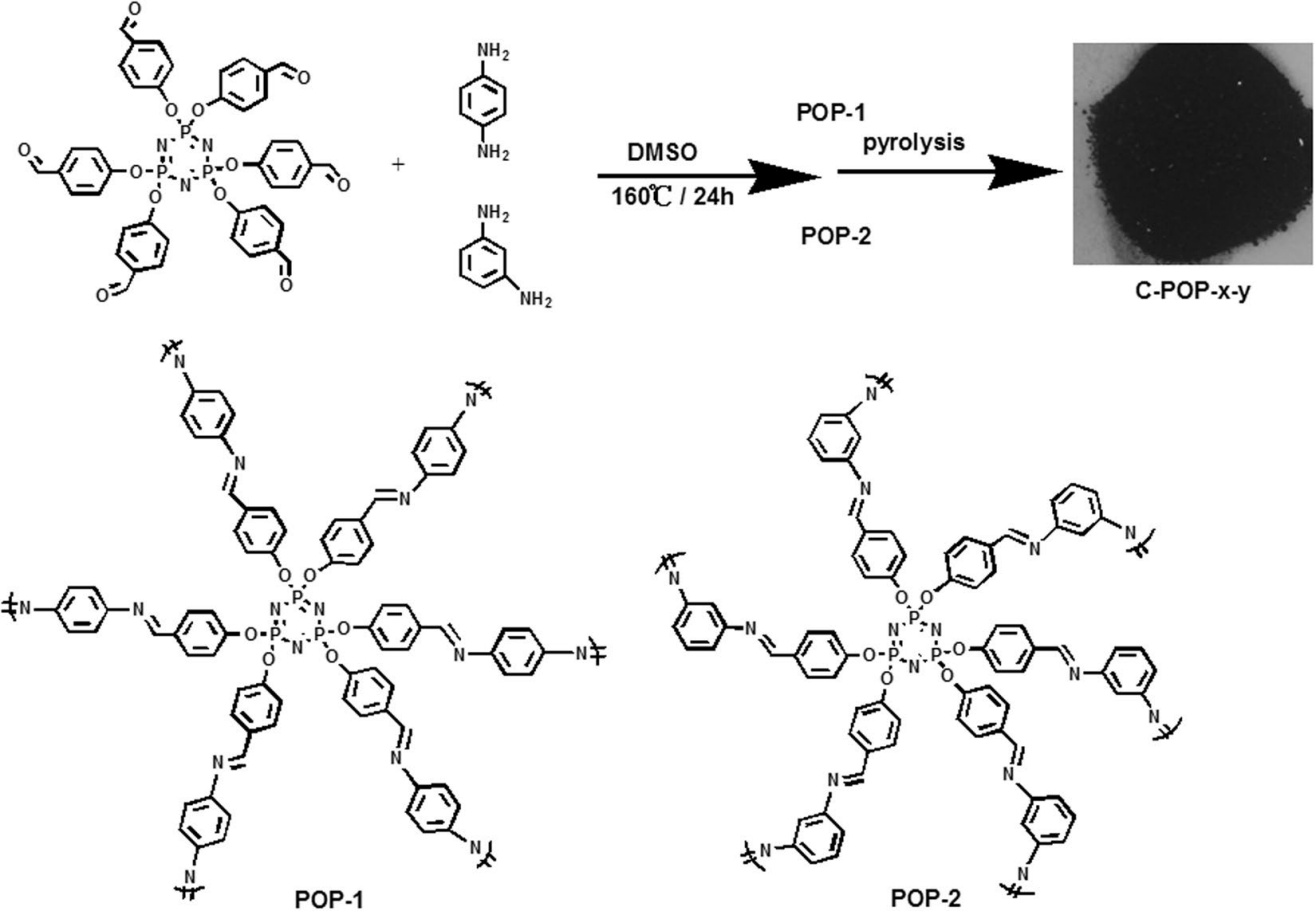
Synthetic route of N, P co-doped carbon networks. For the name C-POP-x-y, x represents monomer while y represents carbonization temperature.

Herein, we present a facile and non-metal involved procedure to prepare N/P dual-doped carbon materials derived from an imine-based porous organic polymer. The precursor imine-based POPs was easily synthesized in one step and with high yield. In this strategy, heteroatom building blocks (i.e. N, P) could be easily introduced to construct a multiple heteroatom-doped POPs, after pyrolysis the multiple heteroatom-doped porous carbon materials could be easily achieved. As a proof of concept, N and P co-doped carbon materials were simply prepared by pyrolysis of a series of imine-linked POPs synthesized via the Schiff base condensation in DMSO under catalyst-free condition (Fig. 1). The N/P dual-doped carbon materials exhibit hierarchical porosity containing micro-, meso−, and macropores with a high surface area of 1535 m^2^ g^−1^. The performance of these materials as cathode catalysts for the ORR was investigated. The optimized catalyst exhibits favorable activity towards ORR with better long-term durability and higher tolerance to methanol crossover than the commercial Pt/C reference electro-catalyst. Therefore, imine-linked porous organic polymers are promising precursor materials for fabrication of novel multiple-heteroatom doped porous carbon materials, which are promising materials for electrochemical applications.

## Results and Discussion

### Characterization of C-POPs

**Figure 2:**
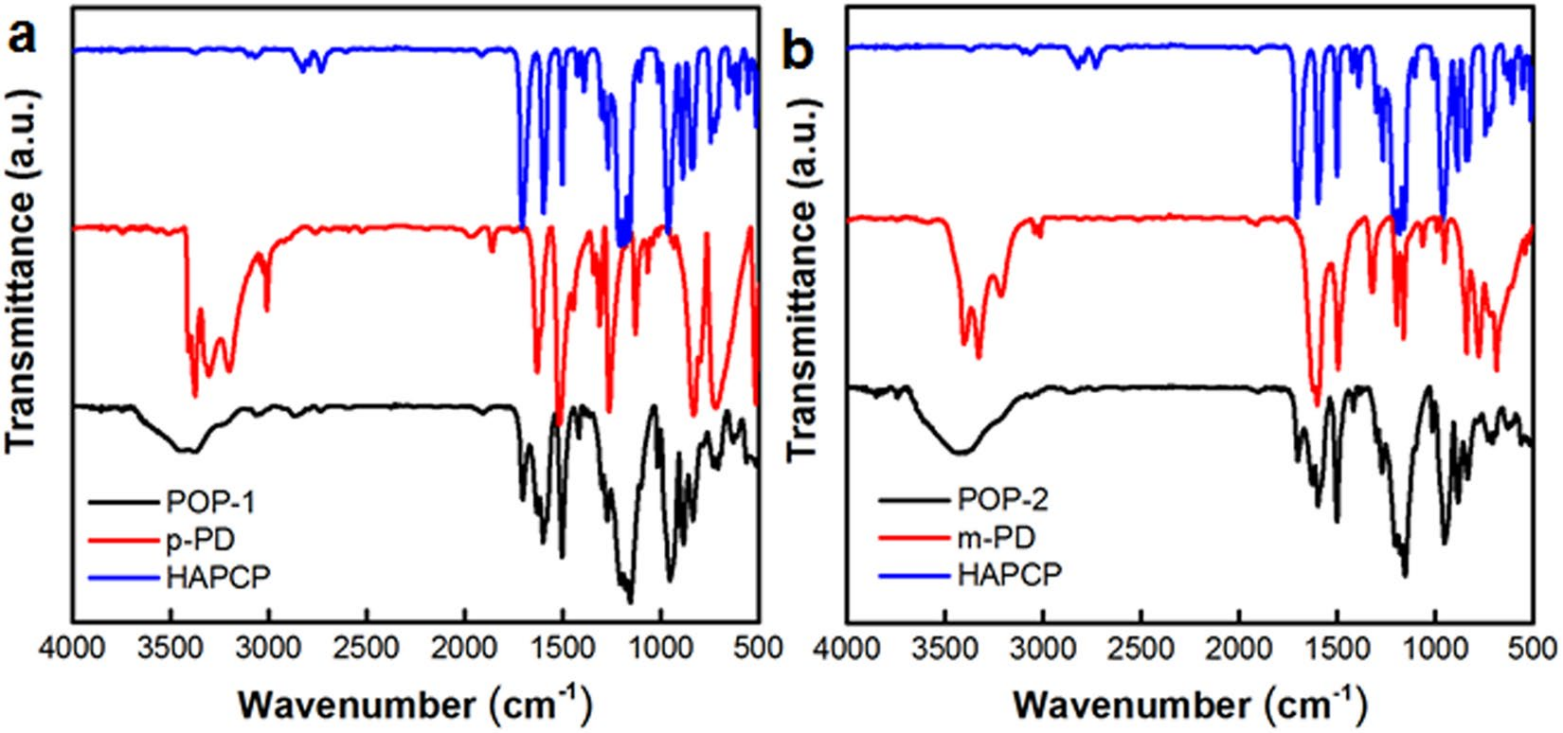
FT-IR spectra of (**a**) the HAPCP (blue), the p-PD (red) and POP-1 (black), (**b**) the HAPCP (blue), the m-PD (red) and POP-2 (black).

The precursors were synthesized via the Schiff base condensation using hexakis(4-formylphenoxy)cyclotriphosphazene (HAPCP) and p-phenylenediamine (p-PD) or m-phenylenediamine (m-PD) (Fig. 1). The imine-linked polymers are denoted as POP-1 and POP-2, respectively. The chemical structures of POPs were confirmed by Fourier transform infrared (FT-IR) spectroscopy (Fig. 2). In FT-IR spectra, bands at 2755–2805 cm^−1^ and ~3345 cm^−1^ which correspond to the stretching vibrations of C-H of aldehyde and -NH_2_ groups of the reactive monomers are largely attenuated in IR spectrum of POP-1 and POP-2, indicating a high degree of polymerization. A fresh peak at 1629 cm^−1^ can be observed clearly, which represents C=N bonds generated from the condensation of aldehyde and amino groups. However, by comparing the IR spectra of HAPCP, there still exists a peak at 1697 cm^−1^ corresponding to C=O vibration in POP-1 and POP-2, which can be attributed to the terminal aldehyde at the edges of the POP-1 or POP-2.

**Figure 3:**
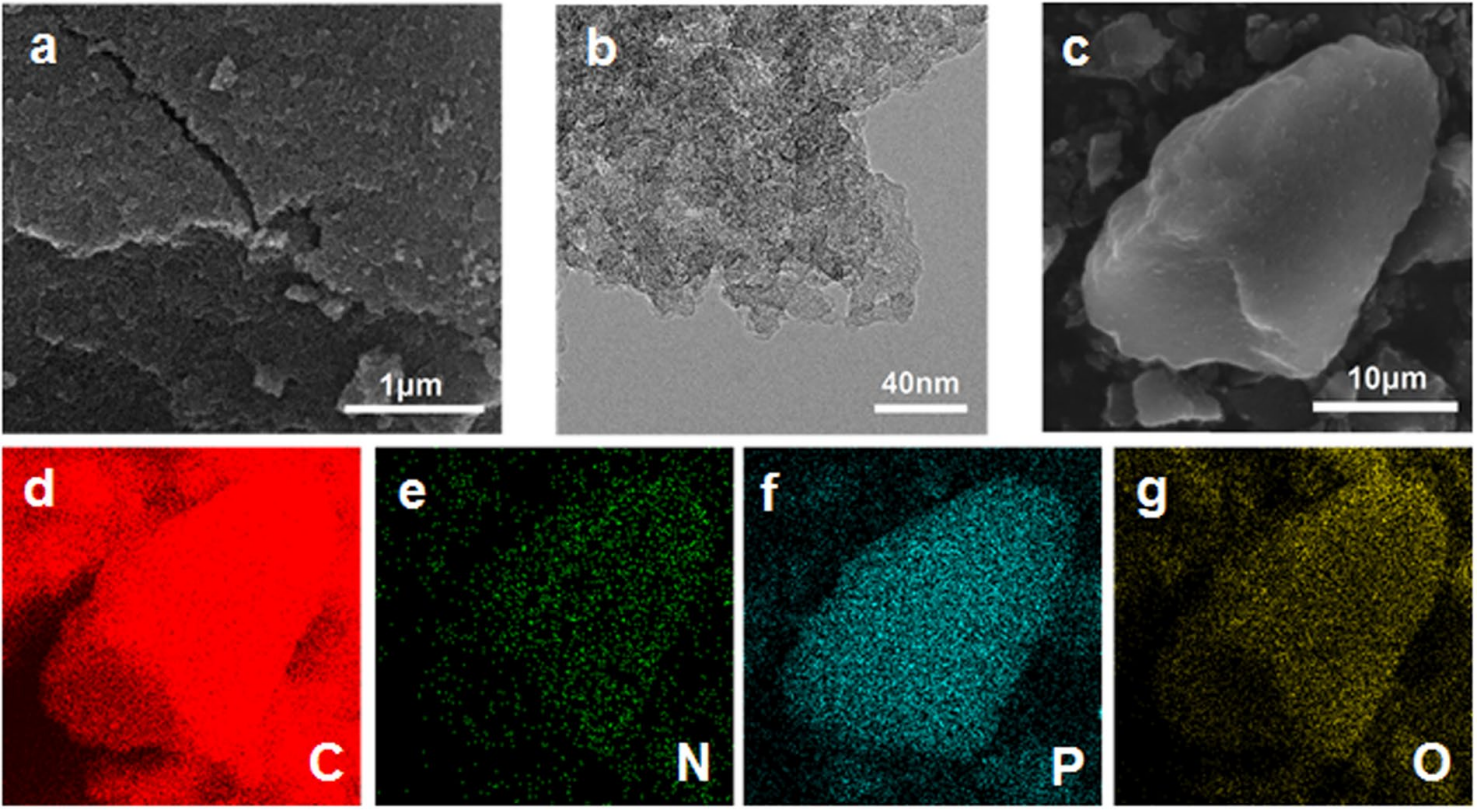
(**a**,**b**) SEM and HR-TEM images of C-POP-2-900, (**c**–**g**) SEM images of C-POP-2-900 with corresponding C, N, P and O elemental mappings.

After pyrolysis of POPs at different temperature, N/P dual-doped carbon networks were obtained and labelled as C-POP-x-y (x: monomer; y: carbonization temperature). The morphology of C-POPs was observed by FE-SEM and HR-TEM (Figs 3 and S1–4). From SEM images, C-POP-1s and C-POP-2s are amorphous bulk materials with rough surface morphology. However, the surface morphology of C-POP-2s is more rough than C-POP-1s (Fig. S1). Such difference may be ascribed to the porous structure of their precursors. As revealed by the nitrogen adsorption measurements, the BET surface area of POP-2 is 344 m^2^ g^−1^ (Fig. S2), while POP-1 shows almost nonporous structure. As a result, POP-2 as precursor is most likely to give rise to carbonized materials with higher surface area. To examine the distribution of heteroatoms, elemental mapping analysis was performed. As shown in Fig. 3d to g, a uniform distribution of C, N, P and O for a sample pyrolyzed at 900 °C (C-POP-2-900) is evident, similar to the C-POP-2-1000 and their precursor before pyrolysis (Figs S3 and S4), indicating homogeneous doping of the heteroatoms. HR-TEM images show that C-POPs mostly consist of disordered carbonaceous products, and parts of ordered arrays of the graphite layers can also be noticed (Figs 3b and S5).

**Figure 4:**
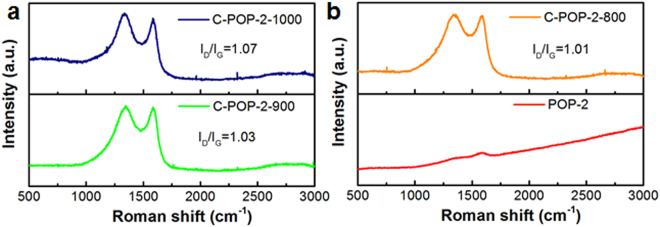
Raman spectra of POP-2, C-POP-2-800, C-POP-2-900 and C-POP-2-1000.

A similar conclusion can also be acquired by the characteristic graphitic peak in Raman spectra (Fig. 4). The curves of carbonized networks display the characteristic D and G bands at roughly 1330 cm^−1^ and 1590 cm^−1^. It has been found that D band reflects crystalline structure disorder, while G band represents in-plane stretching vibration of sp^2^-hybridized carbon atoms^34,46^. The ratio of D-band to G-band intensity (*I*_D_/*I*_G_) gives qualitative information on the graphitic degree in carbon materials. After carbonization, graphitic carbon was formed, which was proven by the presence of graphitic peak in the carbonized materials. Most importantly, the *I*_D_/*I*_G_ intensity ratio of C-POP-2-800, C-POP-2-900 and C-POP-2-1000 are found to be 1.01, 1.03 and 1.07, respectively. With increasing pyrolysis temperature, the *I*_D_/*I*_G_ ratio of C-POP-2 ascend probably due to more defects in graphitic domains derived from gas release under higher temperature pyrolysis.

**Figure 5:**
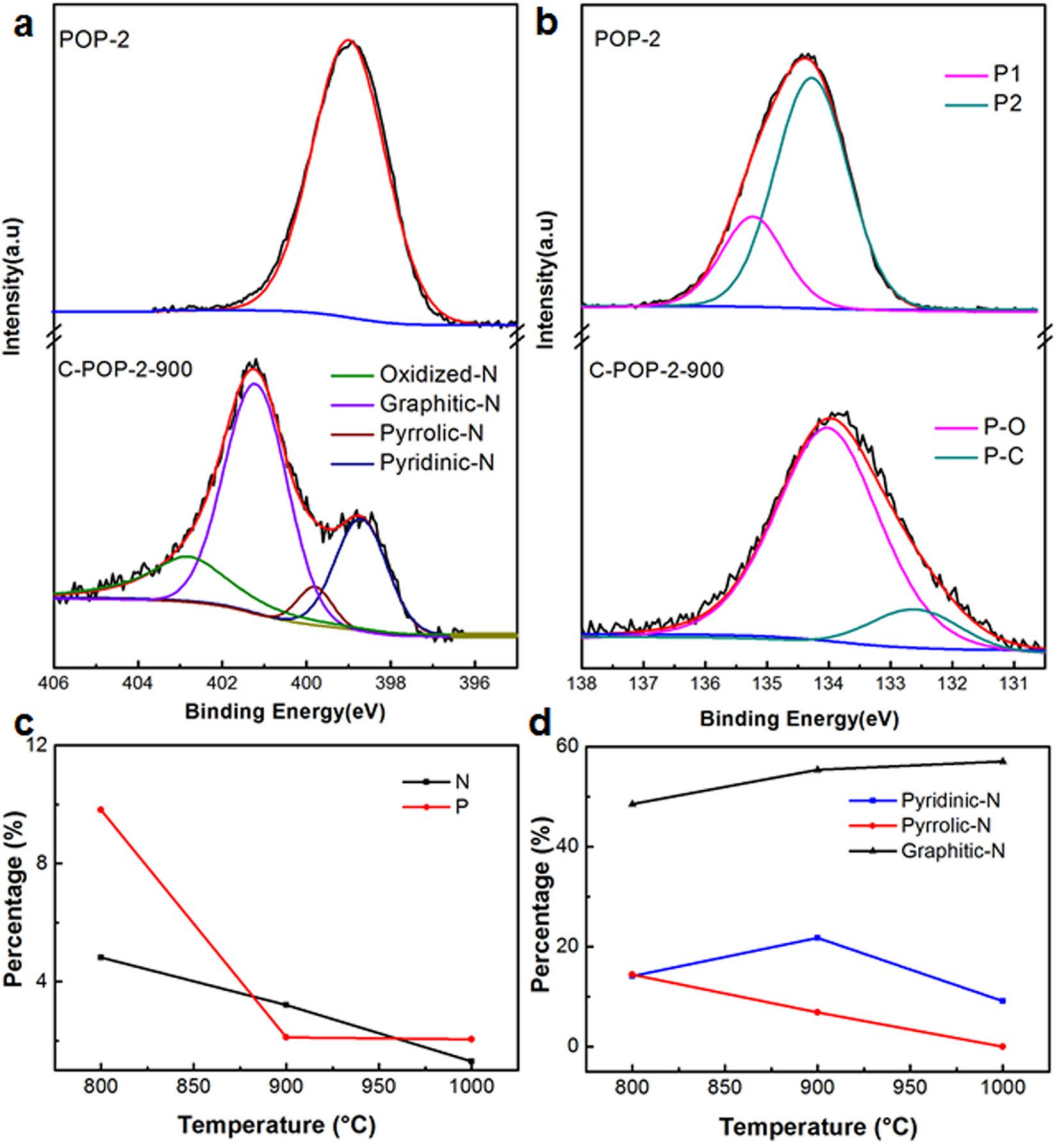
(**a**) XPS N_1s_ spectra of POP-2 and C-POP-2-900, (**b**) P_2p_ spectra of POP-2 and C-POP-2-900. The curve of N1s spectra were fitted to four curves: pyridinic-N, pyrrolic-N, graphitic-N and oxidized-N, (**c**) The percentage contents of N and P of different pyrolysis temperature and (**d**) the corresponding N species contents obtained from the XPS measurements.

X-ray photoelectron spectroscopy (XPS) was performed to investigate the chemical composition and surface property of the carbonized materials. The presence of C, N, P and O indicates the formation of the N/P dual-doped carbon networks (Figs 5, S6 and S7). As revealed by XPS analysis, the C-POP-2-900 has nitrogen and phosphorus contents of 3.21 and 2.12 wt%, respectively. It is further observed that the contents of nitrogen and phosphorus for C-POP-2-800 are 4.82 wt% and 9.82 wt%, while those for C-POP-2-1000 decrease to 1.31 wt% and 2.05 wt%. The results are summarized in Table 1, indicating that the doped heteroatoms contents decrease with increasing carbonization temperature. In the XPS N_1s_ spectra (Fig. 5a), the POP-2 only demonstrates one peak at ~399.0 eV due to a single nitrogen-containing functional group^39,47^. While the pyrolysis resulted in four different N 1s peaks at 398.4 eV, 399.3 eV, 401.2 eV and 402.6 eV, corresponding to the pyridinic-N, pyrrolic-N, graphitic-N, and oxidized-N bonding configurations, respectively (Figs 5a and S6), indicating the decomposition of the POP-2 and the formation of new nitrogen species^33,44,46^. The pyridinic-N and pyrrolic-N are located at the graphitic edges, the former donates one p-electron to the aromatic π system and the latter contributes two p-electrons to the π system. It is reported that relatively increased contents of pyrrolic and pyridinic N sites may be responsible for an improved electrocatalytic activity toward the ORR because their electronic structure benefits the adsorbing and reducing of the O_2_ species via a complete transferred electron reaction^26,48^. Graphitic-N is the nitrogen atom which substitutes carbon atoms and thus incorporated into the graphitic layers. They are considered as ORR catalytic active sites due to the reduced adsorption energy. The changes in pyridinic-N, pyrrolic-N and graphitic-N contents by the pyrolysis temperature are displayed in the Fig. 5d and summarized in Table 1. As a result, the high-temperature pyrolysis induced the decomposition and reconstruction of N species, resulting in the transformation of pyridinic-N and pyrrolic-N to graphitic-N^49^. Although the fact that ORR catalytic activity of carbon can be enhanced by doping N has been proved, the contribution of specific N or other heteroatomic species to the catalytic activity is still unclear or there may be a synergistic effect with nitrogen^32–35^. We further analyzed the phosphorous in these materials. The P_2p_ binding energy (BE) peaks of POP-2 are centered at 134.5 eV, which can be divided into two different bands at ~135.2 eV (P1) and 134.2 eV (P2), corresponding to the core levels of P atoms. After carbonization, the P_2p_ peaks were shifted to 133.9 eV. It can be assigned as the P-C bond (132.5 eV) and the P-O bond (134 eV)^50,51^. The P content seriously decreased when pyrolyzing at 900 °C and kept constant at higher temperature. For P-O containing carbons, the phosphate-like structures bound to carbon lattice, i.e. P-O-C species, showed higher thermal stability than P-C or P-N species, which existed at temperature lower than 800 °C. At higher temperature, the P content dramatically decreased due to the loss of P-C or P-N species and the volatility of elemental phosphorus. The residual P mostly existed as P-O-C species, which was thermally stable at the temperature as high as 1000 °C. Besides, the microporosity was significantly improved at temperature higher than 900 °C, as shown in Table S1. In this case, the location of P species in such ultra-small micropores could effectively hinder their evaporation^52,53^.These results suggest that thermal treatment exerts important influence on determining multidimensional structures and heteroatoms contents.

**Table 1:** The contents of the C, O, N and P for POP-2 and carbon networks and the relative N species analyzed by N 1s XPS spectra of the carbon networks.

Sample	C^a^ (wt%)	O^a^ (wt%)	N^a^ (wt%)	P^a^ (wt%)	Pyridinic-N^b^ (wt%)	Pyrrolic-N^b^ (wt%)	Graphitic-N^b^ (wt%)	Oxidized-N^b^ (wt%)
POP-2	68.99	12.89	10.72	7.40				
C-POP-2-800	71.40	13.96	4.82	9.82	14.05	14.42	48.50	23.03
C-POP-2-900	85.79	8.88	3.21	2.12	21.78	6.88	55.34	15.99
C-POP-2-1000	87.91	8.73	1.31	2.05	9.50	—	57.03	33.47

^a^The total C, O, N, P contents of the samples are considered as 100 wt%, ^b^The weight percentage of the relative N species occupying in the total N content.

**Figure 6:**
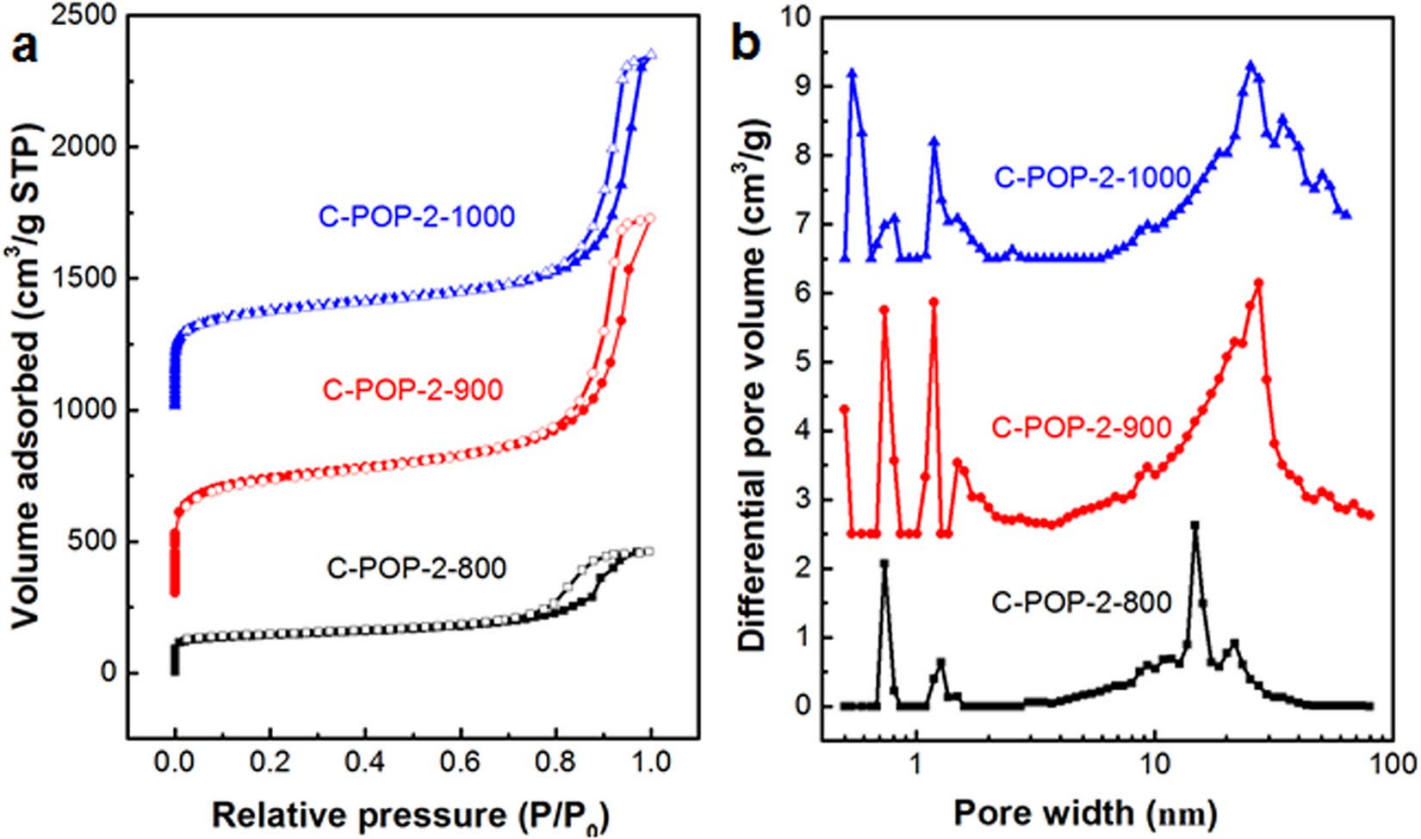
(**a**) Nitrogen gas adsorption-desorption isotherms (in order to facilitate comparison, the isotherms of C-POP-2-900, C-POP-2-1000 were shifted vertically by 300 and 1000 cm^3^ g^−1^, respectively) and (**b**) corresponding pore size distributions of C-POP-2-800, C-POP-2-900, C-POP-2-1000.

To understand the influence of pyrolysis temperature on the formation of porosity in N/P dual-doped carbon networks catalysts, nitrogen sorption analysis of the carbonized materials was performed again (Figs 6, S2 and Table S1). The BET surface areas of C-POP-2-800, C-POP-2-900, C-POP-2-1000, C-POP-1-900, C-POP-1-1000 are 493, 1535, 1305, 268 and 217 m^2^ g^−1^, respectively. Indeed, the samples carbonized from precursor POP-2 show higher BET surface areas than those of the samples pyrolyzed using precursor POP-1. In general, samples having higher surface areas may provide more electro-catalytically active sites, thus enhancing the ORR activity in the kinetically controlled region^26^. It is clearly shown that all the as-prepared materials display type-I nitrogen sorption isotherms with a sharp nitrogen uptake at the low relative pressures (P/P_0_ < 0.001) and a steep rise at high relative pressures (P/P_0_ = 0.8-1.0) (Figs 6a and S2). In particular, the N_2_ adsorption-desorption isotherms of C-POP-2-900 and C-POP-2-1000 indicate a steep nitrogen gas uptake at low relative pressure (P/P_0_ < 0.001), thus confirming the existence of microporous structure. A slight hysteresis loop and a sharp rise at medium and high pressure region can be observed, indicating the presence of both mesopores and macropores in these materials, respectively^54^. The pore size distribution curves further confirm the presence of such hierarchical porous architecture (Fig. 6b). The precursor POP-2 consists of an obviously broad peak between 10 and 100 nm due to its chemical structure. By comparison, the carbonized networks show somewhat remaining of the original POP-2 pore size distribution. The difference of more micropores and less mesopores and macropores may be attributed to high temperature leading to the collapse of the structure and producing plentiful micropores. When the pyrolysis temperature increases from 800 °C to 900 °C, micropore volume content enlarges and pore size decreases as well. While the temperature increases to 1000 °C, micropore volume content decreases. It could be explained that high temperature destroys structure leading to disappearance of some ultra-small micropore. Therefore, annealing temperatures plays a crucial role on the formation of pores, and for POP-2, 900 °C is found to be the optimum condition with the highest surface area. Considering the high BET surface areas and hierarchical porous structure which may lead to efficient mass transportation, the as-prepared heteroatom-doped carbon materials can be used as promising candidates for ORR catalysis^55,56^.

### Electrochemical measurements

**Figure 7:**
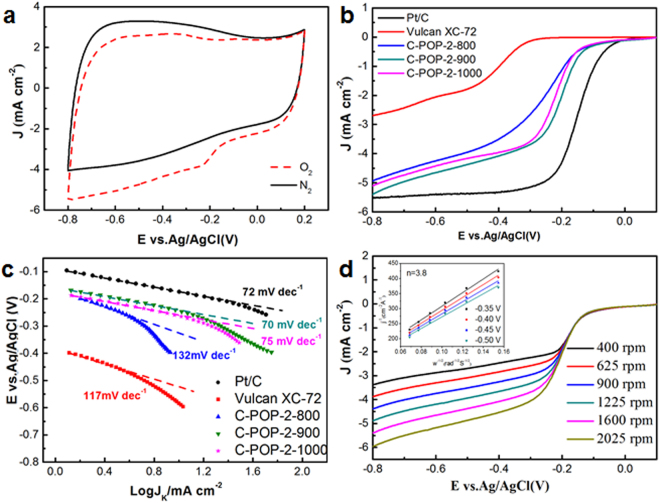
(**a**) CV curves of C-POP-2-900 electrocatalyst in N_2_- and O_2_-saturated 0.1 M KOH solutions at a scan rate of 50 mV s^−1^. (**b**) LSV curves of various electrocatalysts in O_2_-saturated 0.1 M KOH solution at a rotation speed of 1600 rpm and a scan rate of 10 mV s^−1^. (**c**) Tafel plots of the above materials modified electrodes. (**d**) LSV curves of C-POP-2-900 with different RDE rotation speeds in O_2_-saturated 0.1 M KOH at a scan rate of 10 mV s^−1^.

The electrocatalytic properties of the C-POP-2s towards the ORR were first examined by cyclic voltammetry (CV) at a scan rate of 50 mV s^−1^ (Fig. 7a). In contrast to the cyclic voltammograms in N_2_-saturated solution, the well-defined cathodic peaks between −0.8 and 0.2 V (vs Ag/AgCl) are observed in O_2_-saturated 0.1 M KOH, suggesting the dominant ORR process on the surface of C-POP-2s. Among the carbons from different pyrolysis temperature, the C-POP-2-900 shows an ORR peak at the most positive potential (Fig. S8). The linear scan voltammograms (LSV) of different catalysts were collected by the rotating disk electrode (RDE) measurements (Fig. 7b). Comparing to the commercial porous carbon such as Vulcan XC-72, the porous carbon codoped with N and P (C-POP-2-900) exhibits the improved ORR activity with a positive half-wave potential (*E*_1/2_) of −0.19 V, much closer to that of commercial Pt/C (−0.14 V). Further, Tafel plots in the low-overpotential region were presented in Fig. 7c. The diffusion-current-corrected Tafel slope of C-POP-2-900 is calculated to be 70 mV dec^−1^, which is similar to that of commercial Pt/C (72 mV dec^−1^). In contrast, the commercial porous carbon has a much higher Tafel slope as 117 mV dec^−1^, very close to the reported value of non-doping carbon material^57^. It indicates that the N and P co-doping promotes the intrinsic kinetics process of carbon electrode for ORR^58^. According to the XPS results, the pyrolysis at high temperature caused the transformation of the chemical states of N and P elements, generating the dominant percentages of graphitic-N and P-C groups in C-POP-2s. As reported by the literatures, the introduction of N and P atoms altered the charge distribution of adjacent C atoms due to their different electronegativity. The active sites for ORR in the carbon networks will be created by the synergistic electronic effects of N/P dual-doping^33,59^. On the other hand, the electrical conductivity and surface area also play an important role in affecting the ORR activity^60^. Among the carbonized materials, the C-POP-2-800 has the highest proportions of N and P. However, the relatively low pyrolysis temperature cannot efficiently reduce the charge-transfer resistance or bring about the formation of sufficient pores. Although the doped heteroatoms are gradually removed from the carbon networks, the electrical conductivity of C-POP-2s increased with the pyrolysis temperature^33^. Based on the above analysis, the surface area of C-POP-2s dramatically enlarged with the pyrolysis temperature increasing from 800 to 900 °C and then slightly declined when further increasing to 1000 °C. The POPs derived carbon material such as C-POP-2-900 possesses an extremely large surface area of 1535 m^2^ g^−1^, which is composed of hierarchical ordered porous structures. In contrast, the carbon material (M-900), directly by carbonization of the mechanical mixtures of the monomers (HAPCP and m-PD) at 900 °C, possesses a much lower surface area as only 372 m^2^ g^−1^ (Fig. S10 and Table S1). It is found that the M-800 and M-900 exhibit relatively poor electrocatalytic activity toward ORR with relative to the corresponding C-POP-2-800 and C-POP-2-900 materials (Fig. S11). For comparison, the calculation from nitrogen gas adsorption-desorption isotherms of commercial Vulcan XC-72 carbon gives a much lower value of BET surface area as 108 m^2^ g^−1^. The results highlight the specific merits of POPs as carbon precursors, which not only provide the feasibility in introducing the N and P heteroatoms into the carbon network, but also achieve the high specific surface area. The synergistic electronic effects of N/P dual-doping will create more catalytically active sites for ORR in the carbon networks. The high specific surface area is favorable for exposing sufficient contact sites accessible by oxygen as well as meso-microporous connecting channels for the electrolyte/reactant/product diffusion^36,61,62^. Therefore, the highest electrocatalytic performance of C-POP-2-900 can be attributed to the combined effects of N/P co-doping, electrical conductivity, and porous textural properties.

To provide a deep insight into the kinetics of the ORR, the Koutecky-Levich (K-L) plots (*J*^−1^ vs. *ω*^1/2^) were derived from the LSV curves at various rotation speeds. The good linearity of the K-L plots at different reaction potentials indicates the first-order reaction kinetics toward the concentration of dissolved oxygen. The electron transfer number (*n*) can be calculated from the following K-L equation^29^:

1 $$1/{J}=1/{{J}}_{{\rm{k}}}+1/{{J}}_{{\rm{d}}}=1/{{J}}_{{\rm{k}}}+1/({B}{\omega }^{1/2})$$

where *J*, *J*_k_ and *J*_d_ represent the measured, kinetic and diffusion-limiting current density, respectively. *ω* is the angular velocity of the electrode. *B* could be calculated from the slope of K-L plots based on the Levich equation as follows:

2 $${B}=0.62{nFC}{{D}}^{2/3}{{v}}^{-1/6}$$

where *n* is the number of electrons transferred, *F* is the Faraday constant (*F* = 96485 C mol^−1^), *C* is the concentration of O_2_ dissolved in 0.1 M KOH (*C* = 1.2 × 10^−6^ mol cm^−3^), *D* is the diffusion coefficient of O_2_ in the electrolyte (*D* = 1.9 × 10^−5^ cm^2^ s^−1^) and *v* is the kinematic viscosity of the electrolyte (*v* = 0.01 cm^2^ s^−1^).

The average value of *n* was calculated to be approximately 3.8 for C-POP-2-900 from the slope of the K-L plots at different potentials. The result implies that the N/P co-doping and porous architecture favors the four-electron pathway for ORR^63^. For comparison, the values of *n* transferred during ORR on C-POP-2-800 and C-POP-2-1000 were 3.5 and 3.0, respectively (Fig. S9). It indicates that 900 °C is the optimal temperature for such POPs materials to provide a balance of the active sites, electrical conductivity and surface area. The inset in Fig. 7d depicts the corresponding K-L plots for C-POP-2-900 at different potentials.

**Figure 8:**
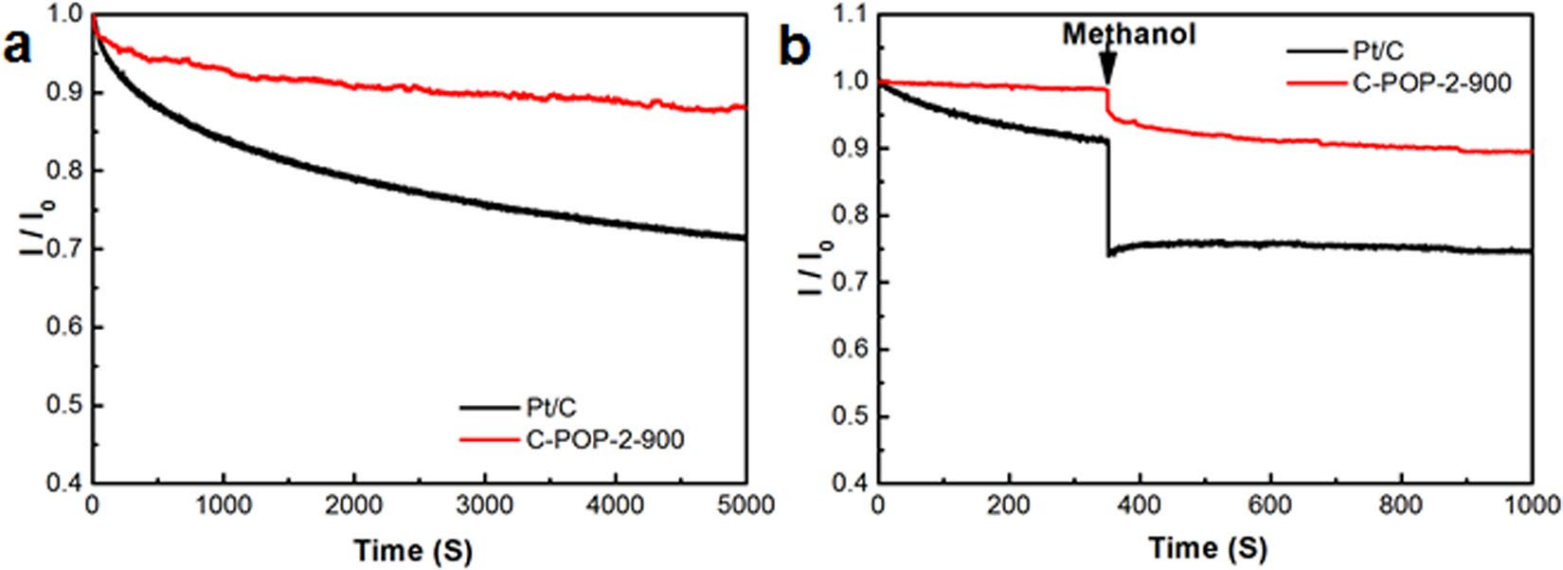
(**a**) Durability evaluation from the *I-t* chronoamperometric and (**b**) Methanol-crossover responses of the C-POP-2-900 electrodes in aqueous solution of KOH (0.1 M) saturated with O_2_ Also included is the commercial Pt/C electrode for comparison.

Considering the practical application of such N/P dual-doping porous carbon materials in direct methanol fuel cells, it is essential to evaluate the durability and methanol crossover effect of the C-POP-2s modified electrode by chronoamperometric (*I-t*) measurements in O_2_-saturated 0.1 M KOH. The commercial Pt/C displayed a sharp slope to 29% activity (Fig. 8a). It is known that the Pt nanoparticles incline to aggregating and detaching from the carbon supports during ORR. In contrast, the C-POP-2-900 presents a better long-term stability with 88% retention of the current density under the same condition. Moreover, the slight current decay after the addition of methanol over C-POP-2-900 catalyst suggests the excellent selectivity toward ORR against methanol oxidation. For comparison, the methanol will pass through the membrane inside the fuel cell and severely competing for the active sites on the Pt/C catalyst. It can be concluded that the metal-free catalysts possess good durability and methanol tolerance superior to the Pt/C in terms of practical operation for fuel cells.

## Conclusion

In summary, we developed a new approach to fabricate multiple heteroatom-doped porous carbon materials based on imine-linked microporous POPs, which are synthesized via a one-step facile strategy by using monomers containing nitrogen and phosphorus elements. Such porous polymers are explored as novel economical precursors to produce N/P dual-doped porous carbon materials. We studied the electrocatalysis performance in oxygen reduction reaction and found that the resultant C-POP-2-900 showed efficient catalytic activities for ORR and good stability. The improved ORR performance of the C-POP-2-900 could be attributed to the electro-catalytically active sites by N/P dual-doping, hierarchical porous carbon nanostructure and relatively large specific surface area. We expect that the strategy could be extended to the development of various heteroatoms multi-doping carbon networks toward versatile energy conversion applications.

## Methods

### Materials and Methods

Dimethyl sulfoxide (DMSO) was freshly dried prior to use. Unless otherwise noted, all other chemicals were purchased and used without further purification. Hexakis(4-formylphenoxy)cyclo-triphosphazene (HAPCP) was synthesized with slight modification of literature method (see Supporting Information)^64,65^. All reactions were carried out under a nitrogen atmosphere.

### Preparation of POPs

Under a nitrogen flow, Hexakis(4-formylphenoxy)cyclotriphosphazene (400 mg) and DMSO (4.0 mL) were added into a 25 mL vessel. After 5 min of stirring, the amine monomer (150.7 mg) dissolved in DMSO (2 mL) was added at the rate of 0.1 mL per min at 50 °C using a syringe pump over 25 min and then the temperature of the oil bath was slowly increased to 160 °C over a period of 20 min. Stop nitrogen flow and the system was in a nitrogen environment. As the temperature of the oil bath reached 160 °C, sign of reflux could be seen inside the flask. Bright yellow to dark-brown solid precipitates were often observed within 10–30 min. The reaction was further kept at 160 °C under nitrogen atmosphere for 24 h. After that the reaction was stopped and cooled down to room temperature, the mixture was filtered and washed three times with anhydrous tetrahydrofuran (THF), washed with tetrahydrofuran in a Soxhlet for 24 h and finally dried in vacuum oven at 60 °C for 24 h.

### General procedure for the preparation of N/P dual-doped carbon materials

A 100 mg of POP-2 was loaded into tube furnace and subsequently heated under an N_2_ atmosphere. The POP-2 powder was first heated at a heating rate of 6 °C min^−1^ up to 300 °C for 3 h and then carbonized at a heating rate of 3 °C min^−1^ up to 900 °C for 1 h. The yield of C-POP-2-900 is around 45%. The products carbonized at different temperature are also obtained and labelled as C-POP-x-y (x: monomer; y: carbonization temperature) following the above procedure.

### General procedure for the preparation of monomeric mixtures based carbon materials

Hexakis(4-formylphenoxy)cyclotriphosphazene (400 mg) and m-phenylenediamine (150.7 mg) were mixed and grinded evenly with a mortar. Then the abrasive powder was pyrolyzed using the conditions which were similar to C-POP-2-900.

### Characterizations

The FT-IR spectra were obtained under ambient conditions in the wavenumber range of 4000–500 cm^−1^ via a Bruker Vertex 70 Spectrometer using the KBr disk method. Raman spectra data were recorded using a 522 nm argon laser with a LabRAM HR800 (HORIBA Scientic, France). The scanning electron microscope (FE-SEM) images and element mappings were collected with a FEI Sirion 200 field emission. The high resolution transmission electron microscopy (HR-TEM) was investigated using a Tecnai G2 F30 microscope (FEI Corp. Holland) operated at 200 kV. The X-ray photoelectron spectroscopy (XPS) data of the samples were carried out via an AXIS-ULTRA DLD-600 W. The pass energy was set at 100 eV and the C 1s line at 284.6 eV was used as a reference. Surface area, N_2_ adsorption isotherms (77 K), pore size distributions were measured using Micromeritics ASAP 2020 M surface area and porosity analyser. The surface areas were calculated from nitrogen adsorption data by Brunauer-EmmettTeller (BET) or Langmuir analysis. Pore size distributions were calculated by DFT methods via the adsorption branch. Before analysis, the samples were degassed at 60 °C for 8 h under vacuum (10–5 bar).

### Electrochemical Tests

Electro-catalytic activities for ORR of the as-prepared catalysts were evaluated by cyclic voltammetry (CV) and rotating disk electrode (RDE) techniques on a CHI-650E electrochemical analyzer. A three-electrode cell was employed using a glass carbon RDE (diameter: 5 mm, area: 0.196 cm^2^, Pine) as working electrode, an Ag/AgCl electrode as the reference electrode and a Pt wire electrode as the counter electrode. To prepare the catalyst ink, 4 mg of the samples was ultrasonically dispersed into 0.8 mL of 2-propanol containing a Nafion solution (5 wt%, DuPont). 18 μL of the catalyst ink was coated on the glassy carbon electrode and dried at room temperature. The commercial Pt/C catalyst was used as reference sample with the Pt loading as 15.28 µg_Pt_/cm^2^. The ORR experiments were carried out in 0.1 M KOH solution. The oxygen reduction reaction was carried out in an O_2_ saturated 0.1 M KOH aqueous solution. The methanol tolerance test was recorded by adding methanol to the O_2_ saturated 0.1 M KOH aqueous solution at around 400 s with a rotation speed of 1600 rpm.

## Electronic supplementary material

Supplementary Information
