# Supplementary material for: A Facile Approach to Prepare Multiple Heteroatom-Doped Carbon Materials from Imine-Linked Porous Organic Polymers
Source: Sci Rep. 2018 Mar 9;8:4200. doi: 10.1038/s41598-018-22507-2 (PMC5844873; doi:10.1038/s41598-018-22507-2)
Supplement: Supplementary file 1 — Supplementary Information [file 41598_2018_22507_MOESM1_ESM.pdf]

Supplementary Information

A Facile Approach to Prepare Multiple Heteroatom-Doped Carbon Materials from Imine-Linked Porous Organic Polymers

Juan Yang, Min Xu, Jingyu Wang,* Shangbin Jin,* and Bien Tan

Key Laboratory of Material Chemistry for Energy Conversion and Storage, Ministry of Education; School of Chemistry and Chemical Engineering, Huazhong University of Science and Technology, Wuhan, 430074, China.

* Correspondence and requests for materials should be addressed to J. Y. and S. J. (E-mail: jingyu.wang@163.com, jinsb@hust.edu.cn)

Contents

1. Synthesis of $[N_3P_3(OC_6H_4CHO)_6]$…………………………………………...S-3
2. Fig. S1 SEM of C-POP-1-900, C-POP-1-1000, C-POP-2-900 and C-POP-2-1000……………………………………………………………..…...S-4
3. Fig. S2 Nitrogen gas adsorption-desorption isotherms of POP-2……………......S-5
4. Fig. S3 SEM and HRTEM images of C-POP-2-1000………..……….……..…..S-6
5. Fig. S4 SEM images of POP-2……………..………………………..……........S-7
6. Fig. S5 HR-TEM of C-POP-2-900 and C-POP-2-1000………………...………..S-8
7. Fig. S6 XPS N1s spectra of C-POP-2-800 and C-POP-2-1000.………………........S-9
8. Fig. S7 XPS survey spectra of POP-2, C-POP-2-800, C-POP-2-900 and C-POP-2-1000 ……………………………………………………………………...S-10
9. Fig. S8 CV curves of C-POP-2-800 (a) and C-POP-2-1000(b) electrocatalysts...S-11
10. Fig. S9 LSV curves of C-POP-2-800 (a) and C-POP-2-1000(b) electrocatalysts.S-12
11. Fig. S10 Nitrogen gas adsorption-desorption isotherms of M-900……..…..…..S-13
12. Fig. S11 Comparison of LSV polarization curves of carbon electrocatalysts from different precursors..…………………………………….………………S-14
13. Fig. S12 Nitrogen gas adsorption-desorption isotherms of Vulcan XC-72……...S-15
14. Table S1. Surface area and porosity of the as-prepared Porous Organic Polymers and Carbonized Products…………..…..………………...……..……S-16
15. Table S2.ORR performance comparison between the C-POP-2-900 and phosphorus or nitrogen-based mesoporous carbons reported recently………………………..S-17
16. References…………………………………………………………..……........S-18

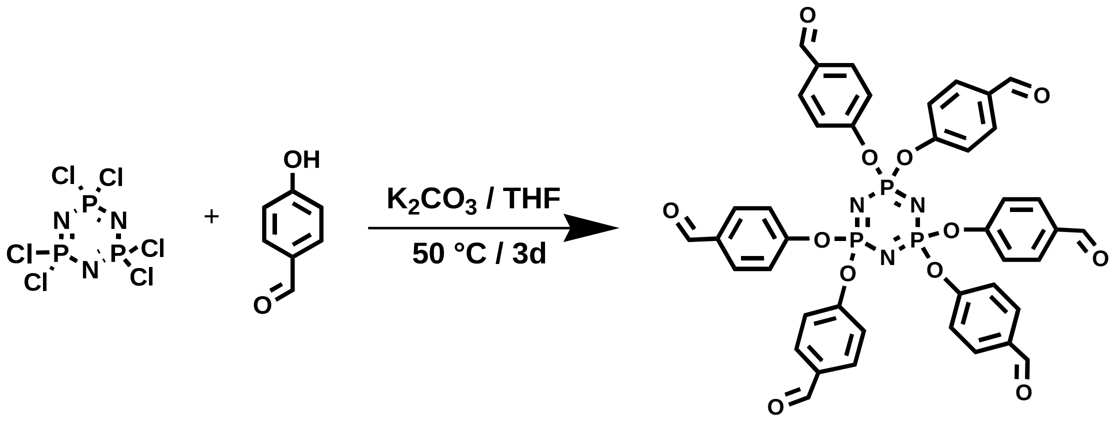

Synthesis of [$N_3P_3(OC_6H_4CHO)_6$]. A mixture of [$N_3P_3Cl_6$] (2.09 g, 6.0 mmol), *p*-Hydroxybenzaldehyde (4.5 g, 36.8 mmol), and K_2CO_3 (10 g, 10.8 mmol) in THF (50 ml) was heated at 50 °C for 3 days under a nitrogen atmosphere. 100 ml pure water was added after the reaction mixture cooling to room temperature. Then the mixture was stirred at room temperature for additional 3 h. The residue was washed with plenty of water. The resulting white solid was dried in vacuo at 60 °C for 48 h. Yield: 4.7 g (90%). ^{1}H NMR (400MHz, $CDCl_3$): δ 9.94 (*s*, 1H, CHO), 7.74 (*d*, 2H, J = 8.5 Hz, H_{ar}) 7.15 (*d*, 2H, J = 8.4 Hz, H_{ar}).

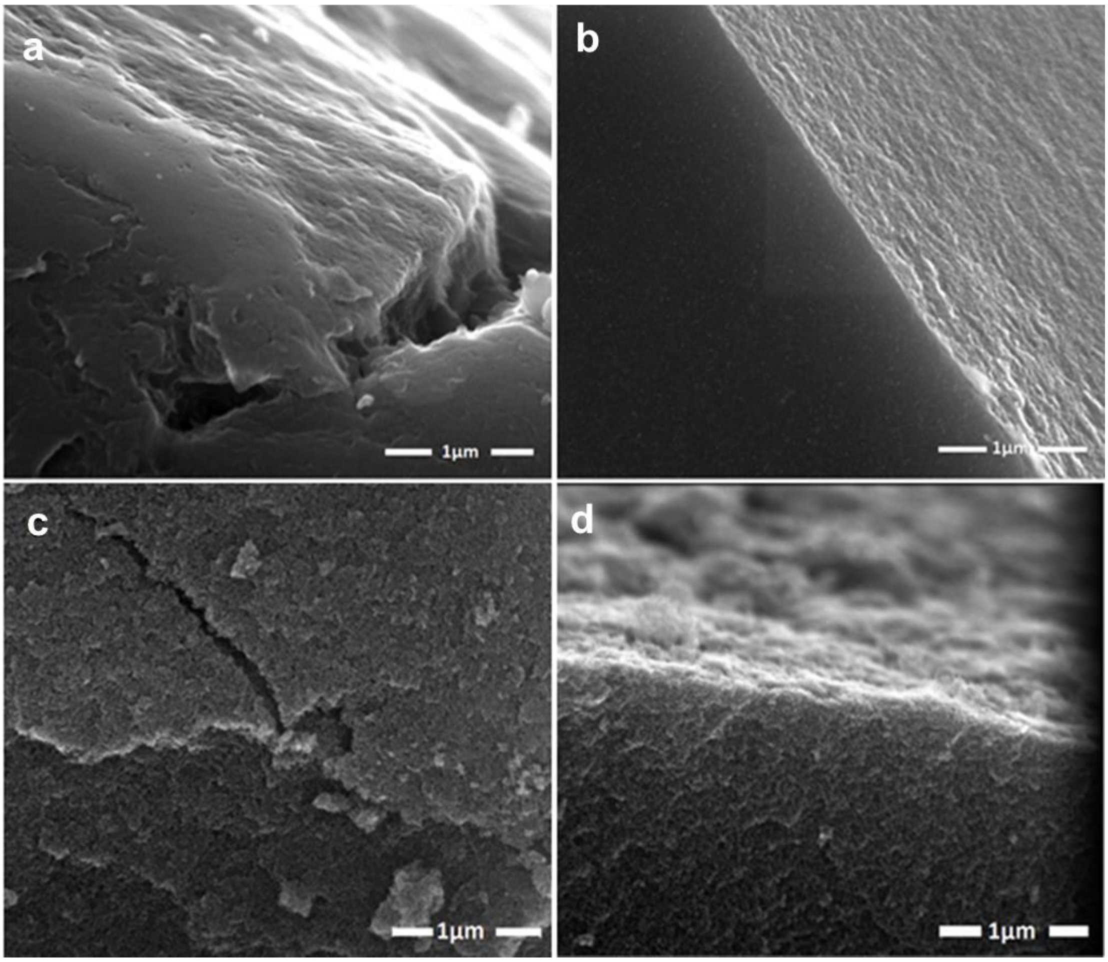

Fig. S1 (a-d) SEM images of C-POP-1-900, C-POP-1-1000, C-POP-2-900 and C-POP-2-1000.

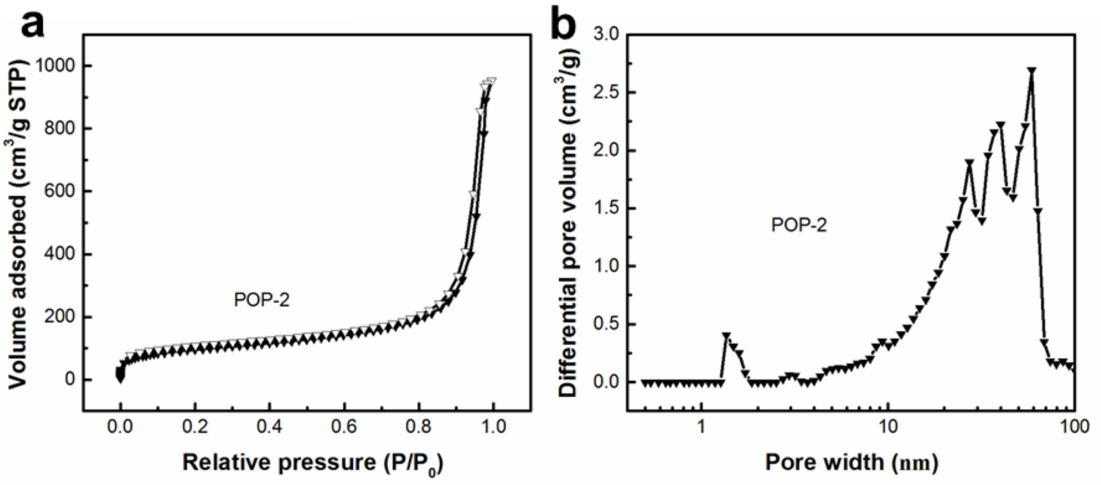

Fig. S2 (a) Nitrogen gas adsorption-desorption isotherms, and (b) corresponding pore size distributions of POP-2.

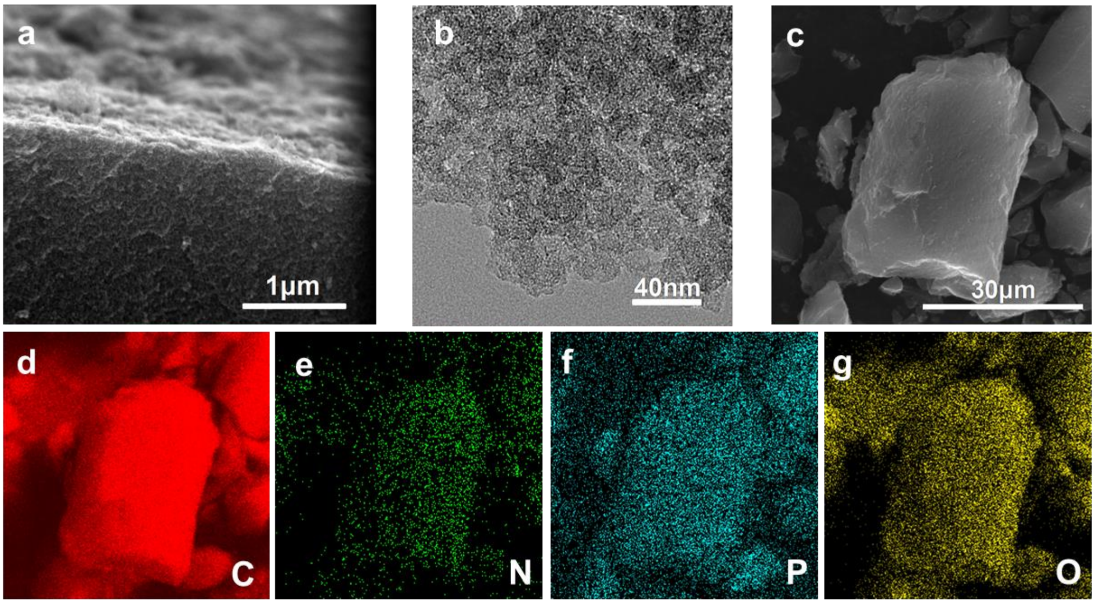

Fig. S3 (a-b) SEM and HRTEM images of C-POP-2-1000, (c-g) SEM images of C-POP-2-1000 with corresponding C, N, P and O elemental mappings.

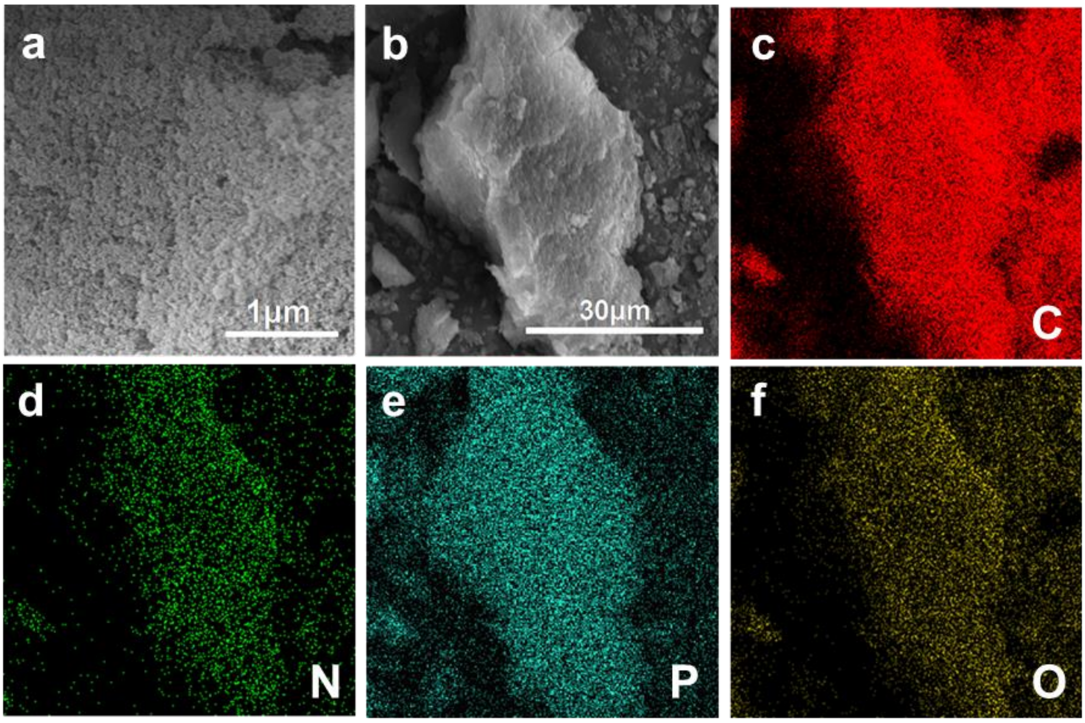

Fig. S4 (a) SEM images of POP-2, (b-f) SEM images of POP-2 with corresponding C, N, P and O elemental mappings.

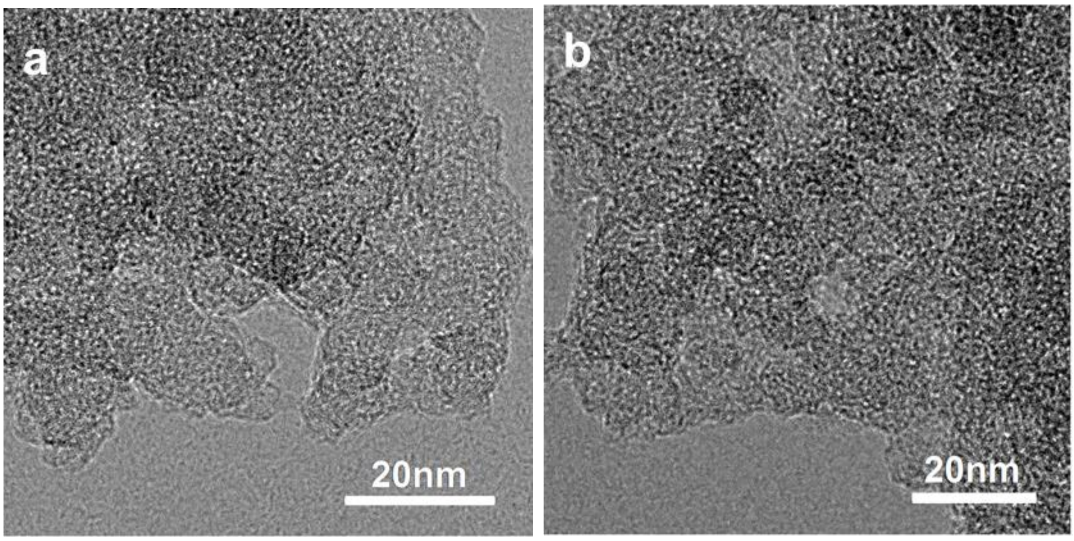

Fig. S5 (a-b) HR-TEM of C-POP-2-900 and C-POP-2-1000.

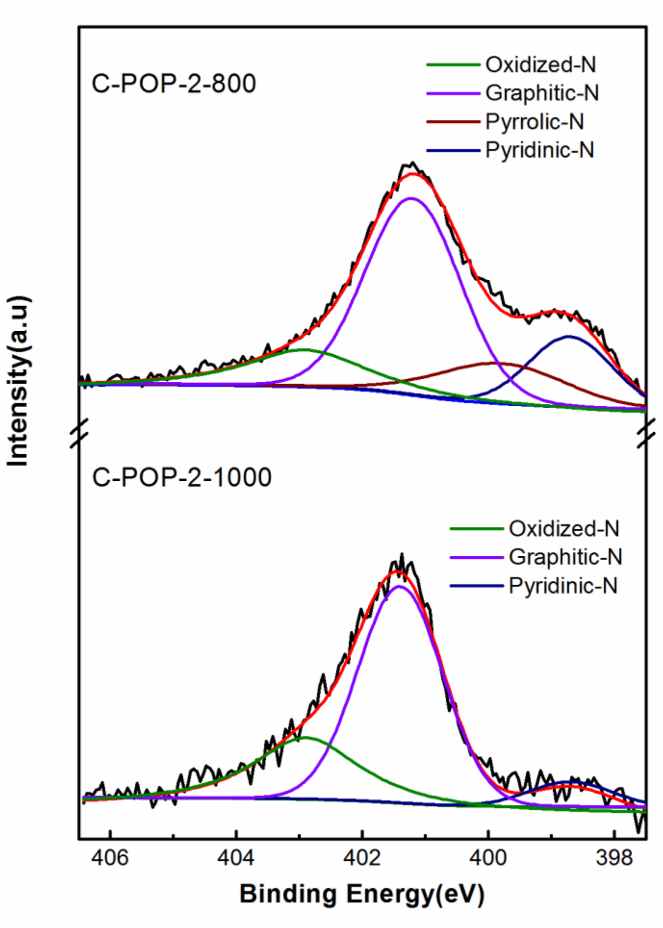

Fig. S6 High resolution XPS N1s spectra of C-POP-2-800 and C-POP-2-1000.

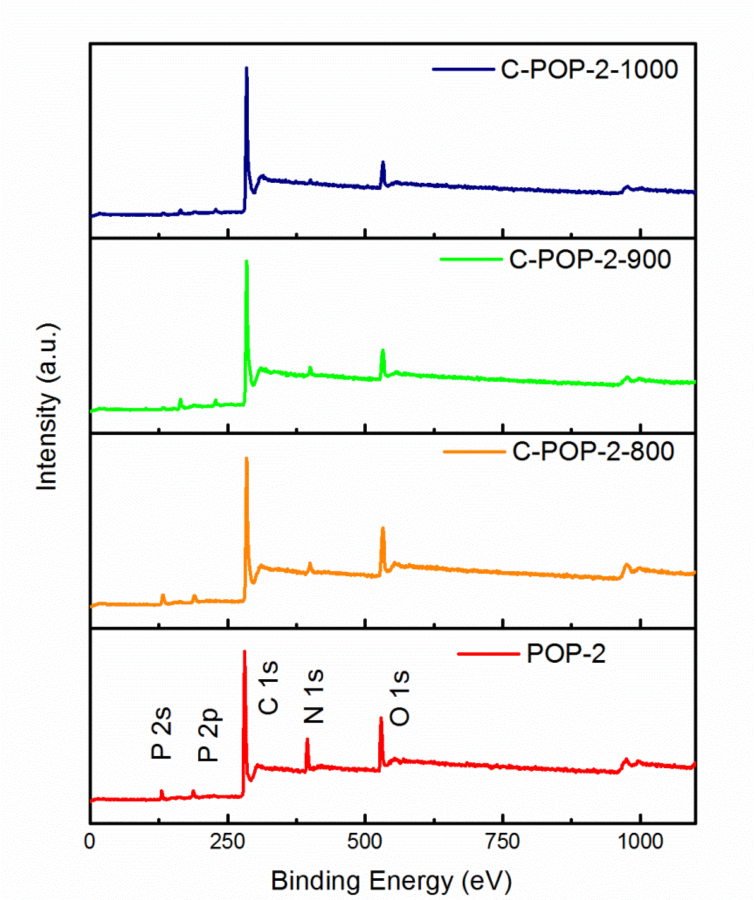

Fig. S7 XPS survey spectra of POP-2, C-POP-2-800, C-POP-2-900 and C-POP-2-1000.

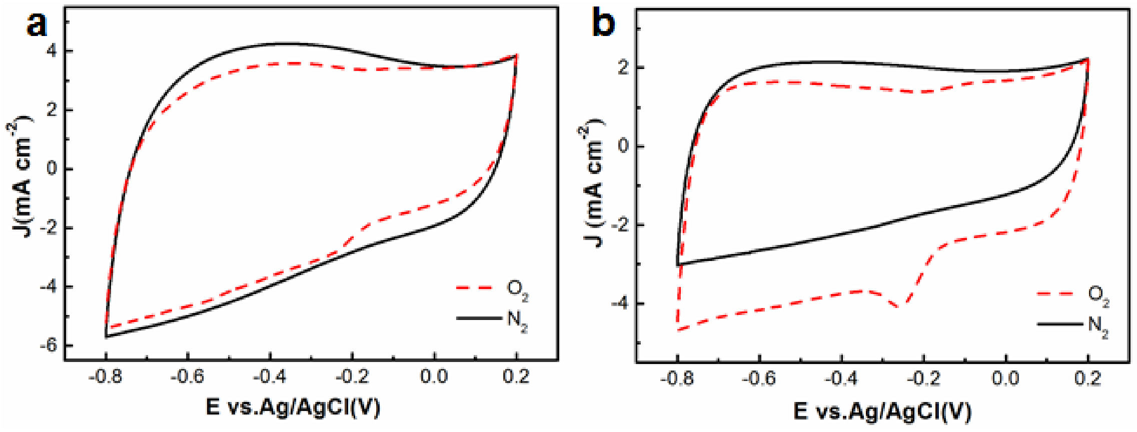

Fig. S8 CV curves of C-POP-2-800 (a) and C-POP-2-1000 (b) electrocatalysts in N_2- and O_2-saturated 0.1M KOH solutions at a scan rate of 50 mV s^{-1}.

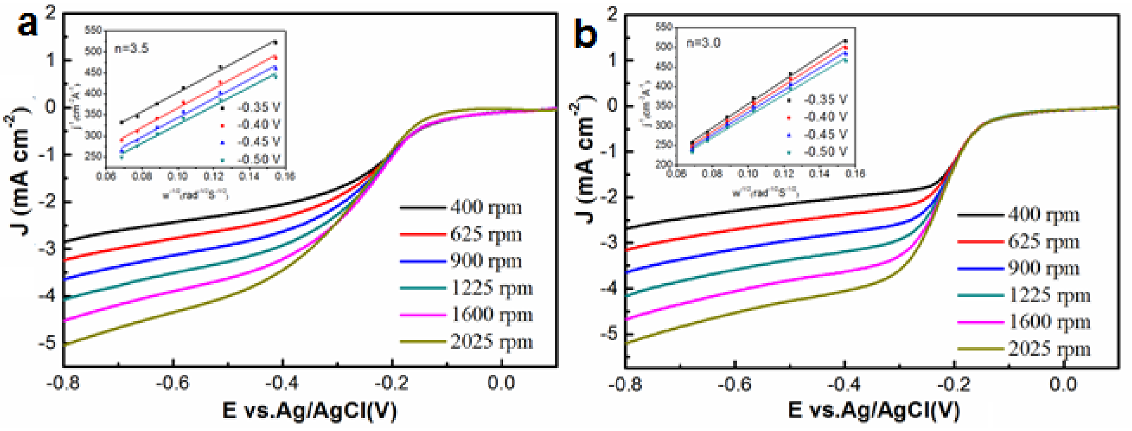

Fig. S9 LSV curves of C-POP-2-800 (a) and C-POP-2-1000 (b) electrocatalysts with different RDE rotation speeds in O_2-saturated 0.1 M KOH at a scan rate of 10 mV s^{-1}. The insets are the corresponding K-L plots at different potentials.

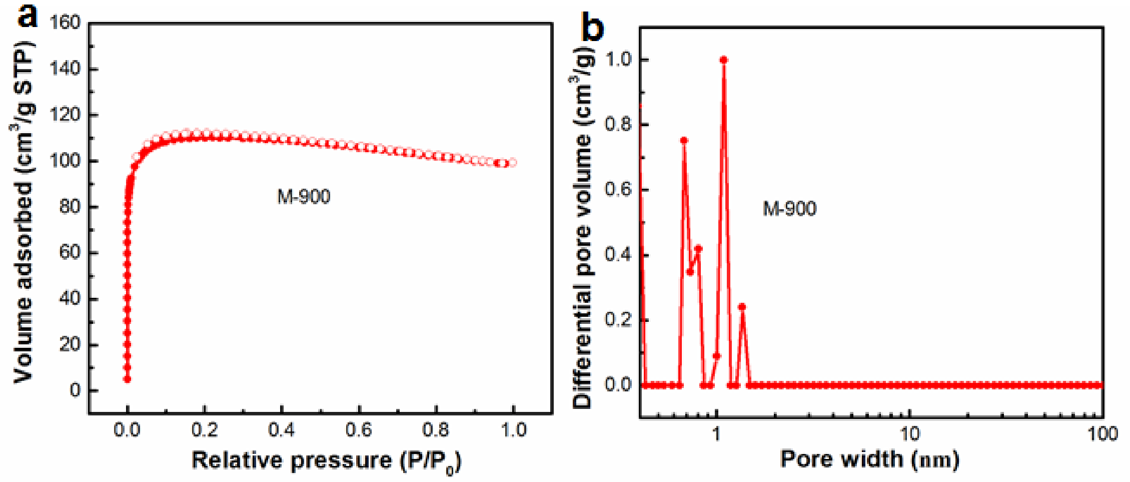

Fig. S10 (a) Nitrogen gas adsorption-desorption isotherms, and (b) corresponding pore size distributions of M-900.

M-900 and M-800 were prepared by carbonizing the grinded monomer mixture at 800 and 900 °C, respectively. (Hexakis(4-formylphenoxy)cyclotriphosphazene (400 mg) and M-phenylenediamine (150.7 mg) were mixed and then grinded evenly with a mortar) The pyrolysis process was similar to C-POP-2-900. M-800 shows almost nonporous structure, while the BET surface area of M-900 is 372 m^2 g^{-1}.

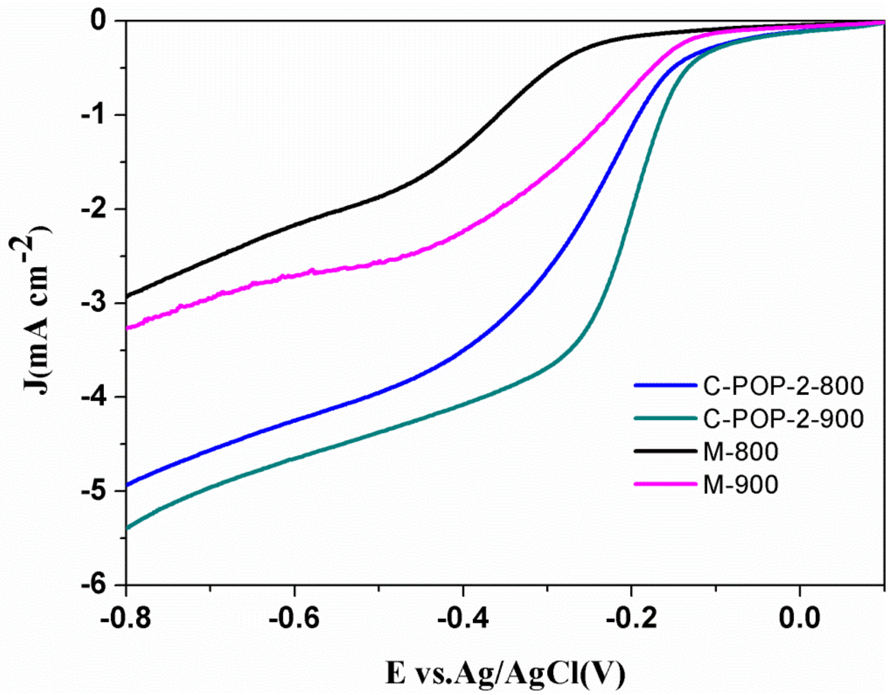

Fig. S11 Comparison of LSV polarization curves of carbon electrocatalysts from different precursors in O_2-saturated 0.1M KOH solution at a rotation speed of 1600 rpm and a scan rate of 10 mV s^{-1}.

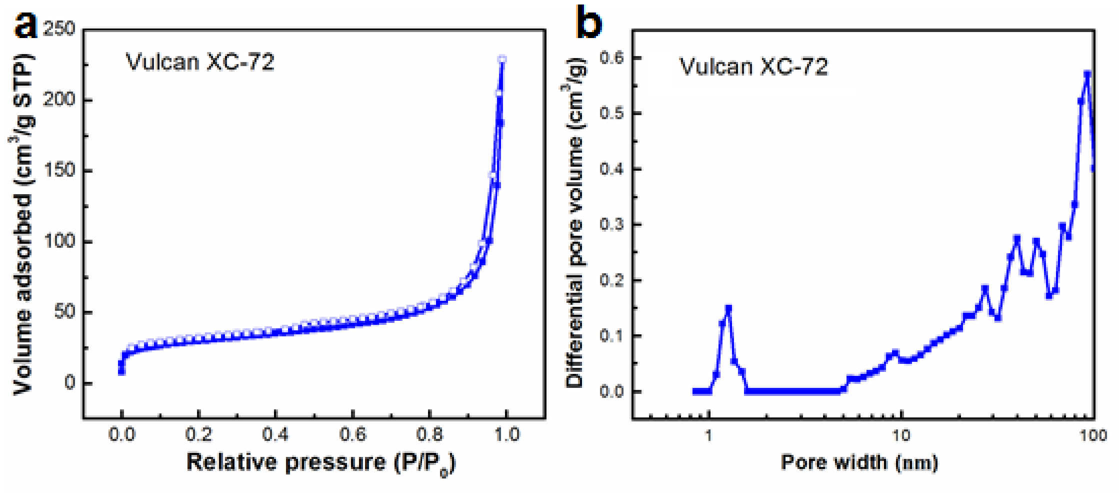

Fig. S12 (a) Nitrogen gas adsorption-desorption isotherms, and (b) corresponding pore size distributions of Vulcan XC-72.

Table S1. Surface Area and porosity of the As-Prepared Porous Organic Polymers and Carbonized Products.

Sample	S_{BET} [a] (m^2/g)	S_L [b] (m^2/g)	PV [c] (cm^3/g)	M.A. [d] (m^2/g)	MPV [e] (cm^3/g)	MPV [f] (%)
POP-2	345	469	1.47	64	0.025	1.70
C-POP-2-800	493	655	0.712	301	0.138	11.6
C-POP-2-900	1535	2051	2.21	866	0.39	17.6
C-POP-2-1000	1305	1746	2.09	711	0.32	15.3
M-900	372	489	0.155	339	0.155	100
Vulcan XC-72	108	122	0.35	40	0.016	4.57

[a]Surface area calculated from N_2 adsorption isotherms at 77.3 K using BET equation. [b]Surface area calculated from N_2 adsorption isotherms at 77.3 K using Langmuir equation. [c]Pore volume calculated from N_2 isotherm at P/P_0=0.995, 77.3 K. [d]t-Plot micropore area. [e]t-Plot micropore volume. [f]t-Plot micropore volume / Pore volume×100 %.

Table S2. Comparison of ORR performance between the C-POP-2-900 and phosphorus or nitrogen-based mesoporous carbons reported recently.

Catalyst	Onset potential (V)	Half-wave potential (V)	Electron transfer number (n)	Electrolyte	Ref.
N-doped graphene	-0.155 (V vs Ag/AgCl)	-0.28 (V vs Ag/AgCl)	3.28	0.1M KOH	1
N-doped mesoporous carbon	-0.11 (V vs Ag/AgCl)	-0.24 (V vs Ag/AgCl)	3.4	0.1M KOH	2
P-doped graphene	0.92 (V vs RHE)	0.58 (V vs RHE)	3.8	0.1M KOH	3
N/S-Codoped Mesoporous Carbon	N/A	−0.24 V(V vs Ag/AgCl)	3.4	0.1M KOH	4
N,P co-doped mesoporous carbon	0.94 (V vs RHE)	0.85 (V vs RHE)	4.0	0.1M KOH	5
N,P dual-doped carbon	0.2 (V vs Ag/AgCl)	-0.2 (V vs Ag/AgCl)	3.5	0.1M KOH	6
N-P-O co-doped 3D Graphene	0.928 (V vs RHE)	0.836 (V vs RHE)	3.83	0.1M KOH	7
N,P-codoped porous carbon	/	-0.14 (V vs Ag/AgCl)	3.8	0.1M KOH	8
N,P-doped carbon	-0.02 (V vs Ag/AgCl)	-0.21 (V vs Ag/AgCl)	3.7	0.1M KOH	9
C-POP-2-900	-0.11 (V vs Ag/AgCl)	-0.19 (V vs Ag/AgCl)	3.8	0.1M KOH	This work

References:

1. Farzaneh, A., Saghatoleslami, N., Goharshadi, E.K., Gharibi, H. & Ahmadzadeh, H. 3-D mesoporous nitrogen-doped reduced graphene oxide as an efficient metal-free electrocatalyst for oxygen reduction reaction in alkaline fuel cells: Role of π and lone pair electrons. *Electrochimica Acta* **222**, 608-618 (2016).
2. Tang, J. et al. Synthesis of nitrogen-doped mesoporous carbon spheres with extra-large pores through assembly of diblock copolymer micelles. *Angewandte Chemie International Edition* **54**, 588-593 (2015).
3. Zhang, C., Mahmood, N., Yin, H., Liu, F. & Hou, Y. Synthesis of phosphorus-doped graphene and its multifunctional applications for oxygen reduction reaction and lithium ion batteries. *Advanced materials* **25**, 4932-4937 (2013).
4. Zhang, S. et al. Protic-Salt-Derived Nitrogen/Sulfur-Codoped Mesoporous Carbon for the Oxygen Reduction Reaction and Supercapacitors. *ChemSusChem* **8**, 1608-1617 (2015).
5. Zhang, J., Zhao, Z., Xia, Z. & Dai, L. A metal-free bifunctional electrocatalyst for oxygen reduction and oxygen evolution reactions. *Nature nanotechnology* **10**, 444-452 (2015).
6. Liu, Q. et al. Cellulose-derived nitrogen and phosphorus dual-doped carbon as high performance oxygen reduction catalyst in microbial fuel cell. *Journal of Power Sources* **273**, 1189-1193 (2015).
7. Zhao, Y. et al. NPO co-doped high performance 3D graphene prepared through red phosphorous-assisted "cutting-thin" technique: A universal synthesis and multifunctional applications. *Nano Energy* **28**, 346-355 (2016).
8. Pan, F., Duan, Y., Liang, A., Zhang, J. & Li, Y. Facile Integration of Hierarchical Pores and N, P-Codoping in Carbon Networks Enables Efficient Oxygen Reduction Reaction. *Electrochimica Acta* **238**, 375-383 (2017).
9. Borghei, M. et al. Porous N, P-doped carbon from coconut shells with high electrocatalytic activity for oxygen reduction: Alternative to Pt-C for alkaline fuel cells. *Applied Catalysis B: Environmental* **204**, 394-402 (2017).
